# Production and structural characterization of eco-friendly bioemulsifier SC04 from *Saccharomyces cerevisiae* strain MYN04 with potential applications

**DOI:** 10.1186/s12934-023-02186-z

**Published:** 2023-09-07

**Authors:** Yasmina A. Elsaygh, Mona K. Gouda, Yasser Elbahloul, Moustafa Abdel Hakim, Nancy M. El Halfawy

**Affiliations:** 1 Botany and Microbiology Department, Faculty of Science, Alexandria University, Moharam Bek, Alexandria, 21511 Egypt; 2 Alexandria Company for Petroleum Additives (ACPA), Alexandria, Egypt

**Keywords:** *Saccharomyces cerevisiae*, Bioemulsifier, Mannoprotein, Stability, Plackett‒Burman (PB), Central composite design (CCD), Biomedical applications

## Abstract

**Background:**

Bioemulsifiers are natural or microbial-based products with the ability to emulsify hydrophobic compounds in water. These compounds are biodegradable, eco-friendly, and find applications in various industries.

**Results:**

Thirteen yeasts were isolated from different sources in Alexandria, Egypt, and evaluated for their potential to produce intracellular bioemulsifiers. One yeast, isolated from a local market in Egypt, showed the highest emulsification index (EI_24_) value. Through 26S rRNA sequencing, this yeast was identified as *Saccharomyces cerevisiae* strain MYN04. The growth kinetics of the isolate were studied, and after 36 h of incubation, the highest yield of cell dry weight (CDW) was obtained at 3.17 g/L, with an EI_24_ of 55.6%. Experimental designs were used to investigate the effects of culture parameters on maximizing bioemulsifier SC04 production and CDW. The study achieved a maximum EI_24_ of 79.0 ± 2.0%. Furthermore, the crude bioemulsifier was precipitated with 50% ethanol and purified using Sephadex G-75 gel filtration chromatography. Bioemulsifier SC04 was found to consist of 27.1% carbohydrates and 72.9% proteins. Structural determination of purified bioemulsifier SC04 was carried out using Fourier transform infrared spectroscopy (FTIR), scanning electron microscopy-energy dispersive X-ray spectroscopy (SEM-EDX), high-performance liquid chromatography (HPLC), and nuclear magnetic resonance spectroscopy (NMR). FTIR spectroscopy revealed characteristic bands associated with carboxyl and hydroxyl groups of carbohydrates, as well as amine groups of proteins. HPLC analysis of monosaccharide composition detected the presence of mannose, galactose, and glucose. Physicochemical characterization of the fraction after gel filtration indicated that bioemulsifier SC04 is a high molecular weight protein-oligosaccharide complex. This bioemulsifier demonstrated stability at different pH values, temperatures, and salinities. At a concentration of 0.5 mg/mL, it exhibited 51.8% scavenging of DPPH radicals. Furthermore, in vitro cytotoxicity evaluation using the MTT assay revealed a noncytotoxic effect of SC04 against normal epithelial kidney cell lines.

**Conclusions:**

This study presents a new eco-friendly bioemulsifier, named SC04, which exhibits significant emulsifying ability, antioxidant and anticancer properties, and stabilizing properties. These findings suggest that SC04 is a promising candidate for applications in the food, pharmaceutical, and industrial sectors.

## Introduction

Surface active agents are among the most produced compounds worldwide, as they have a crucial role in interfacial and surface tension reduction between two immiscible liquids [1]. These substances are essentially classified into two groups: low-molecular-weight surfactants and high-molecular-weight emulsifiers [2]. Glycolipids, phospholipids or lipopeptides are the chemical components of low-molecular-weight surfactants, while high-molecular-weight emulsifiers consist of amphipathic polysaccharides, proteins, lipopolysaccharides, lipoproteins, or biopolymers mixed with the previously mentioned compounds [2, 3]. Additionally, these molecules can efficiently emulsify two immiscible liquids, such as hydrocarbons or other hydrophobic substrates, even at low concentrations [4]. The effectiveness of bioemulsifiers is dependent on their chemical composition and the number of reactive groups exposed in their structure [4, 5]. Furthermore, they are characterized by low toxicity, environmental compatibility, biodegradability, and high efficiency under extreme conditions (pH, temperature, and salinity) [6, 7]. Various organisms, such as yeasts, filamentous fungi, and bacteria, can produce bioemulsifiers with different molecular structures [8]. Indeed, *S. cerevisiae* is a bioemulsifier producer and offers the advantage of not being toxic; therefore, it is generally regarded as safe (GRAS) [7].

Bioemulsifiers have drawn attention due to their advantageous properties over synthetic emulsifiers, which enable them to become prominent in various industrial and environmental applications [4]. These applications include the formation of stable emulsions in the food and cosmetics industries, textiles, and pharmaceuticals [9]. They improve the consistency of fat-soluble vitamins, fatty acids, and amino acids [10]. Moreover, they are also referred to as “green molecules” due to their widespread use in contaminated soil bioremediation or other environmental pollution [11]. Bioemulsifiers have been extensively researched for potential applications in the petroleum industry, such as microbial-enhanced oil recovery (MEOR) [12].

*S. cerevisiae* cell walls are the main source of mannoproteins with interesting emulsifying properties [13]. Moreover, yeast-derived mannoproteins have health-promoting properties due to their aliphatic structure [14]. Despite the various potential benefits of bioemulsifiers, they face challenges, including low-yield production. Therefore, the current study emphasizes the isolation of *S. cerevisiae* with potent emulsifying activity and optimization to maximize the yield and large-scale production. Extraction, purification, and characterization of the new bioemulsifier in terms of stability, chemical structure, and physicochemical and biological properties were also investigated in this study.

## Results

### Morphological characterization and identification of a yeast isolate

**Fig. 1:**
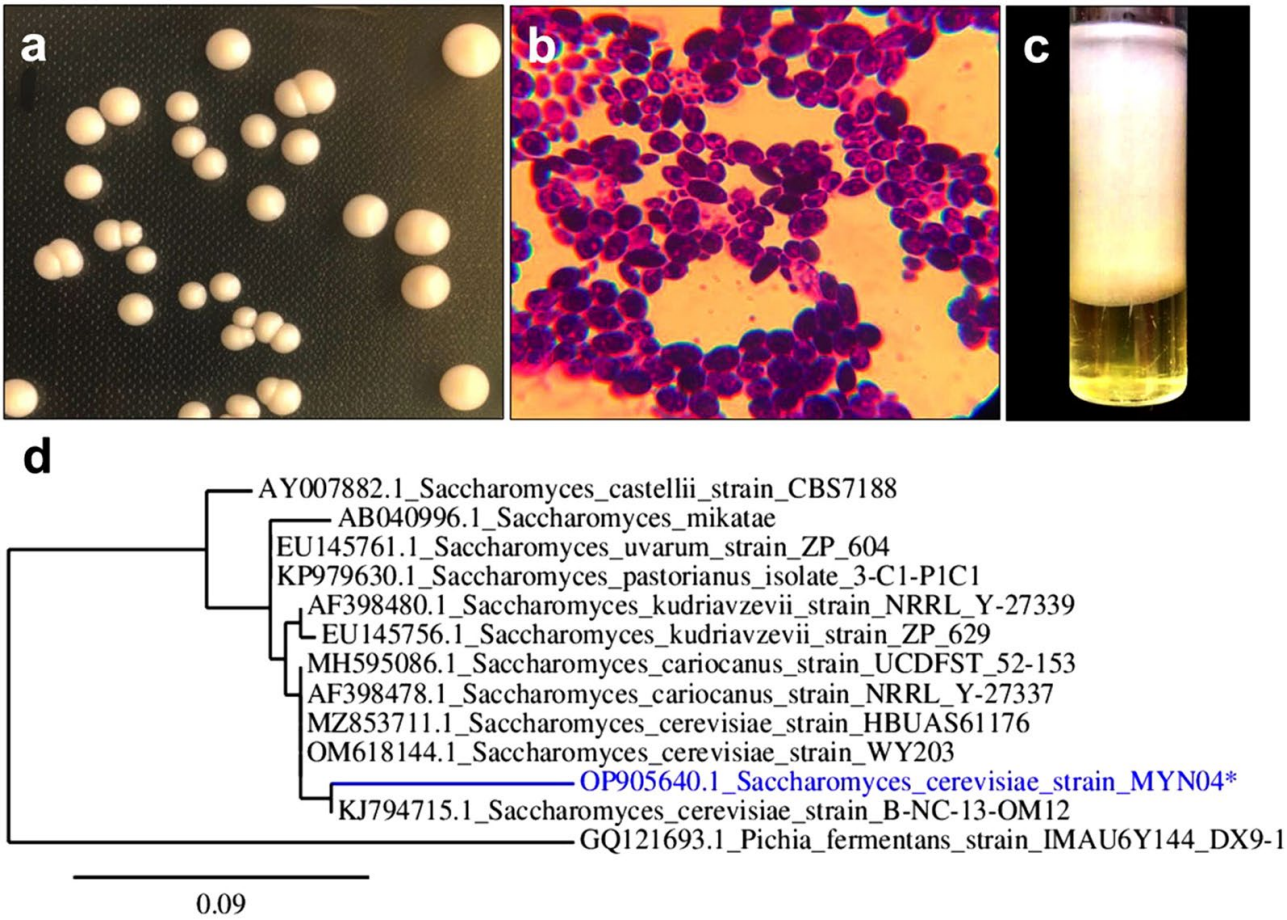
**a** Colony morphology of *S. cerevisiae* strain MYN04 cultivated on yeast malt (YM) agar, **b** yeast isolate morphology under a light microscope at 1000 × magnification, **c** emulsion formed after adding 4 mL of the supernatant to 6 mL kerosene after 24 h, and **d** phylogenetic analysis of *S. cerevisiae* strain MYN04, showing its relationships with reference strains in the NCBI database. The scale bar indicates a genetic distance of 0.09

Among thirteen isolated yeasts, one yeast (isolated from an Egyptian local market) revealed the highest EI_24_ and was selected for further investigation. The yeast isolate was identified as *S. cerevisiae* using 26S rRNA gene amplification and deposited in the NCBI GenBank database under the accession number OP905640. Microscopy revealed that the strain possessed a dispersed coccoidal cell and that the colony morphology was smooth. Phylogenetic analysis based on the 26S rRNA gene sequence revealed the evolutionary relationships for the yeast isolate MYN04 with respect to other closely related *S. cerevisiae* strains (Fig. 1).

### Kinetics of bioemulsifier production

*S. cerevisiae* strain MYN04 was cultivated on Cooper and Paddock’s medium at 30 °C with shaking at 150 rpm. Cells were allowed to grow for different time intervals as follows: 12, 24, 36, 48, 60, and 72 h. The maximum cell dry weight (CDW; 3.2 g/L) and EI_24_ (55.6%) were recorded after 36 h of incubation. This incubation period was selected for further experiments.

### Single-factor optimization of bioemulsifier production

The effect of different carbon and nitrogen sources on bioemulsifier biosynthesis in the MYN04 strain was investigated. The maximum CDW (3.1 g/L) and EI_24_ (58.72%) were achieved using sucrose as the sole carbon source. Furthermore, yeast extract revealed the highest CDW (2.9 g/L) and EI_24_ (59.6%) as the sole nitrogen source.

### Statistical optimization of bioemulsifier biosynthesis

**Fig. 2:**
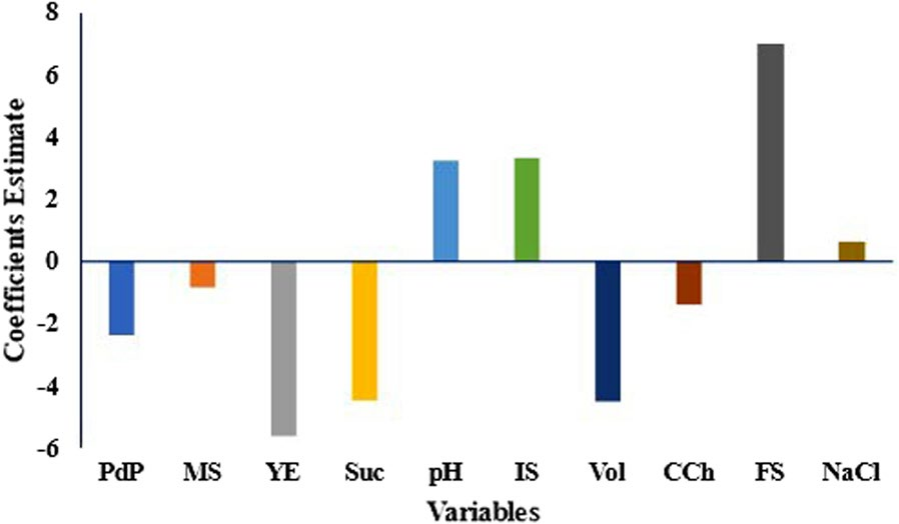
Effects of culture conditions on the biosynthesis of bioemulsifier by *S. cerevisiae* strain MYN04, estimated from the coefficients of the Plackett‒Burman design. *PdP* KH_2_PO_4_, *MS* MgSO_4_, *YE* yeast extract, *Suc* sucrose, *IS* inoculum size, *Vol* medium volume, *CCh* CaCl_2_, *FS* FeSO_4_

Factors influencing bioemulsifier biosynthesis by *S. cerevisiae* strain MYN04 were screened using PB Design. Maximum EI_24_ values of 64.6% and 62.0% were observed in runs 2 and 7, respectively. Both runs were performed at a pH value of 7.0, 25.0 g/L sucrose, and 0.04 g/L FeSO_4_ (Table 1). EI_24_ was observed at a pH value of 3.0, although the FeSO_4_ concentration was 0.04 g/L (Table 1). The correlation coefficient (R^2^) for the model is 0.992, and the adjusted R^2^ is 0.949, indicating the suitability of the model applied in the PB design. The model was significant (*P* = 0.043), and sucrose, FeSO_4_, volume of medium, and yeast extract were the significant variables. The concentration of FeSO_4_ was shown to positively influence the biosynthesis of the biosurfactant by MYN04 strain, along with the pH value and the size of the inoculum. The other factors negatively affected the biosynthesis of the biosurfactant (Fig. 2). On the other hand, the growth of *S. cerevisiae* strain MYN04 was significantly influenced by all studied variables except dipotassium hydrogen phosphate and calcium chloride. The maximum CDW obtained was 3.9 g/L (data not shown).

**Table 1:** Statistical screening of factors influencing the biosynthesis of bioemulsifier by *S. cerevisiae* strain MYN04 via the PB

Run #	KH_2_PO_4_	MgSO_4_	Yeast Extract	Sucrose	pH	Inoculum Size	Volume	CaCl_2_	FeSO_4_	NaCl	EI_24_, %
1	1.5	2.5	7	25	3.0	3	125	0.3	0.01	0.1	36.5
2	0.5	7.5	3	25	7.0	1	125	0.3	0.04	0.1	64.6
3	1.5	7.5	7	15	3.0	1	125	0.1	0.04	1.0	32.7
4	0.5	7.5	7	25	3.0	1	75	0.3	0.01	1.0	43.1
5	1.5	2.5	3	15	7.0	1	125	0.3	0.01	1.0	57.7
6	0.5	2.5	3	15	3.0	1	75	0.1	0.01	0.1	44.0
7	1.5	2.5	7	25	7.0	1	75	0.1	0.04	0.1	62.0
8	1.0	5.0	5	20	5.0	2	100	0.2	0.025	0.55	52.8
9	1.0	5.0	5	20	5.0	2	100	0.2	0.025	0.55	52.8
10	1.5	7.5	3	25	7.0	3	75	0.1	0.01	1.0	43.1
11	0.5	2.5	7	15	7.0	3	75	0.3	0.04	1.0	58.5
12	1.5	7.5	3	15	3.0	3	75	0.3	0.04	0.1	55.6
13	0.5	2.5	3	25	3.0	3	125	0.1	0.04	1.0	48.1
14	1.0	5.0	5	20	5.0	2	100	0.2	0.025	0.55	56.6
15	0.5	7.5	7	15	7.0	3	125	0.1	0.01	0.1	57.7

For the optimization of the bioemulsifier synthesized by the CCD, sucrose, ferrous sulphate concentrations, and pH were chosen based on their effects and investigated using the CCD. The resulting emulsification ranged from 43.5 to 72.0%, and four experimental runs (7, 16, 19, and 24) resulted in emulsification from 60 to 72%. The maximum emulsification was obtained by using 20.0 g/L sucrose, 0.05 g/L FeSO_4_, and a pH value of 6.5 (Table 2).

**Table 2:** Optimization of the biosynthesis of bioemulsifier by *S. cerevisiae* strain MYN04 using CCD

Run #	Sucrose, g/L	FeSO_4_	pH	EI_24_, %
1	50	0.03	7.5	45.8
2	38	0.05	6.5	55.0
3	26	0.03	7.5	52.0
4	50	0.07	5.5	50.0
5	50	0.07	7.5	43.5
6	26	0.07	5.5	55.0
7	26	0.07	7.5	64.0
8	26	0.03	5.5	45.0
9	38	0.05	6.5	57.0
10	50	0.03	5.5	47.4
11	38	0.05	6.5	55.0
12	50	0.03	7.5	47.6
13	26	0.03	7.5	52.0
14	50	0.07	7.5	47.0
15	26	0.03	5.5	45.0
16	26	0.07	5.5	60.0
17	50	0.03	5.5	47.1
18	50	0.07	5.5	50.0
19	26	0.07	7.5	65.2
20	38	0.05	6.5	57.0
21	38	0.05	8.0	47.4
22	38	0.05	6.5	55.0
23	38	0.08	6.5	55.9
24	20	0.05	6.5	72.0
25	38	0.05	5.0	48.6
26	38	0.02	6.5	47.4
27	38	0.05	6.5	57.0
28	56	0.05	6.5	55.6

**Fig. 3:**
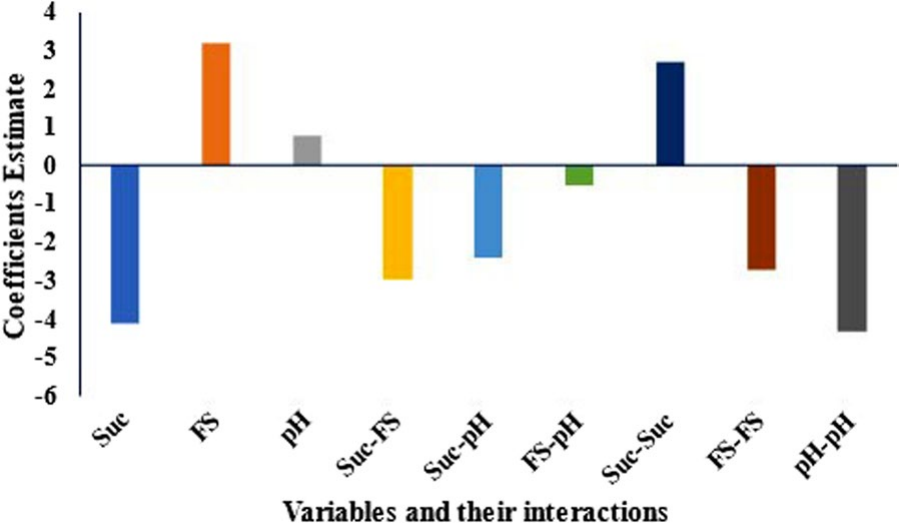
Effects of culture conditions on the biosynthesis of bioemulsifier by *S. cerevisiae* strain MYN04, estimated from the coefficients of the central composite design. *Suc* sucrose, *FS* FeSO_4_

The CCD coefficient estimates revealed that FeSO_4_, pH, and the change in sucrose concentration had positive effects on the biosynthesis of the bioemulsifier. In contrast, increasing sucrose concentration and its interaction with FeSO_4_ and pH had negative effects on the emulsification capabilities of *S. cerevisiae* strain MYN04 (Fig. 3). Analysis of variance of the CCD results indicates that sucrose and FeSO_4_ were significant variables and their interactions. The correlation coefficient (R^2^) for the model is 0.958, and the adjusted R^2^ is 0.935, indicating the suitability of the quadratic model applied (*P* = 0.0001), while the lack of fit was insignificant (*P* = 0.364) (data not shown).

**Fig. 4:**
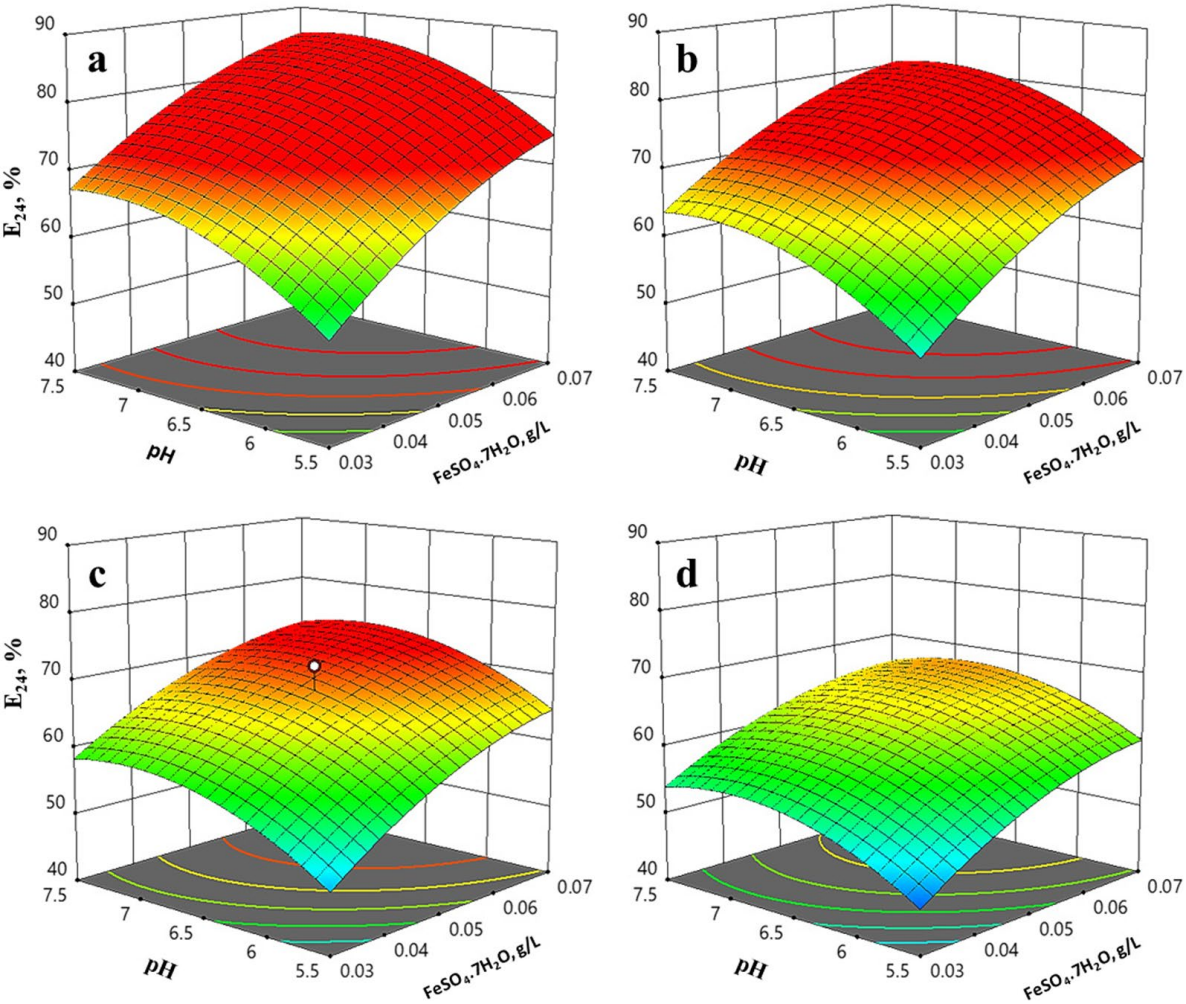
Surface plots showing the effect of pH, ferrous sulphate, and different concentrations of sucrose on the EI_24_ of the bioemulsifier synthesized by *S. cerevisiae* strain MYN04 using the central composite design. Sucrose concentrations: **a** 12.0 g/L, **b** 15.0 g/L, **c** 20.0 g/L, **d** 25.0 g/L

The surface plot reveals the interaction between different variables, highlighting the curvature of the surface. Figure 4a–d illustrates the negative effect of increasing sucrose concentration on the emulsification percentage. The emulsification index reached its maximum at 20.0 g/L sucrose. Furthermore, the positive effect of pH and FeSO_4_ is significantly apparent at lower sucrose concentrations.

Experiments were conducted using optimized values of variables estimated from the model to confirm the validity of the applied model (Table 3). An emulsification value of 79.0 ± 2.0% was obtained, which falls within the prediction interval. The repeated model values were very close to the model predicted EI_24_%, indicating the suitability and accuracy of the model.

**Table 3:** Validation and point prediction of emulsifier biosynthesis by *S. cerevisiae* strain MYN04 applying the CCD quadratic model

Run#	Sucrose (g/L)	Ferrous sulphate (g/L)	pH	EI_24_, %^a^	
				Experimental	Model estimated
19	26.0	0.07	6.5	64.6 ± 0.86	64.7
24	20.0	0.05	6.5	74.0 ± 2.83	69.4
Point prediction	12.0	0.08	7.0	79.0 ± 2.03	77.6–82.3^b^

^a^Results represent the mean and standard deviation

^b^Low and high 95% confidence

### Effect of temperature, salinity, and pH on bioemulsifier stability

**Fig. 5:**
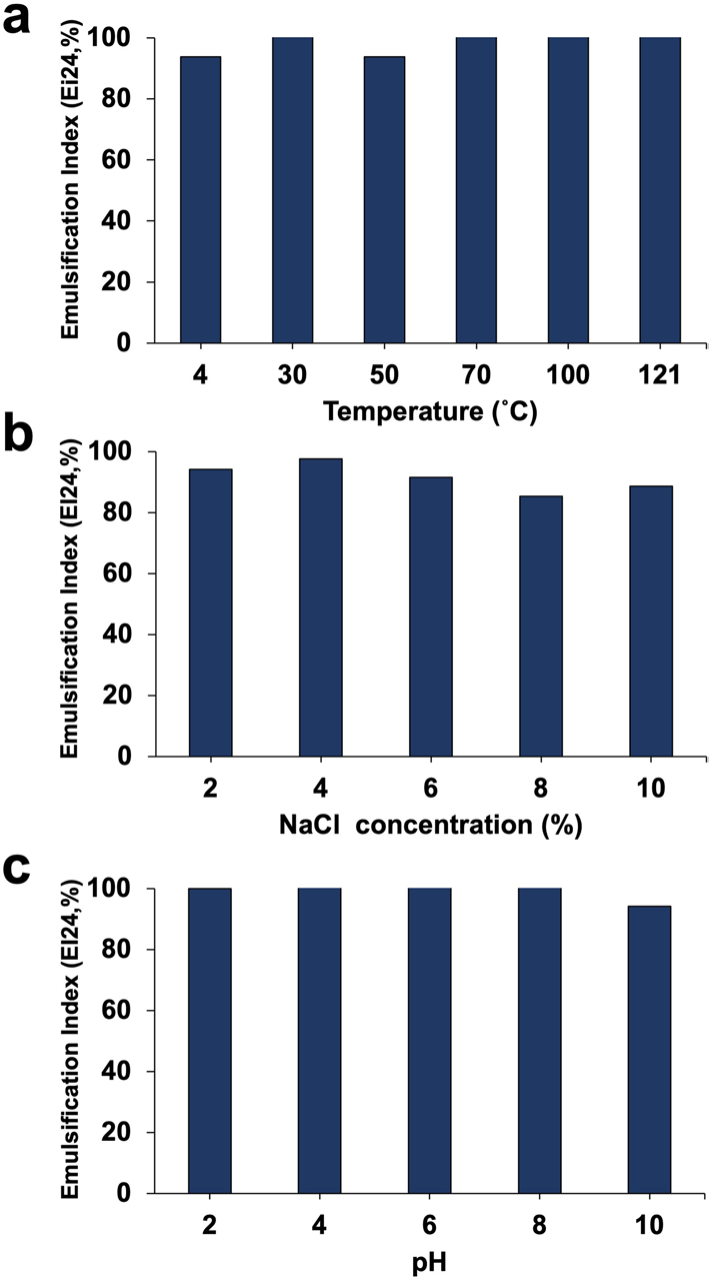
Stability of the bioemulsifier produced by *S. cerevisiae* strain MYN04 at different **a** temperatures, **b** salinities and **c** pH values

The stability and activity of the bioemulsifier were assessed under various environmental conditions (Fig. 5). The results indicated that the bioemulsifier was stable at temperatures of 4, 30, 50, 70, 100, and 121 °C, salinity levels ranging from 2 to 10%, and pH levels ranging from 2 to 10, with an EI_24_% greater than 80%.

### Emulsifying capacity of the bioemulsifier

**Fig. 6:**
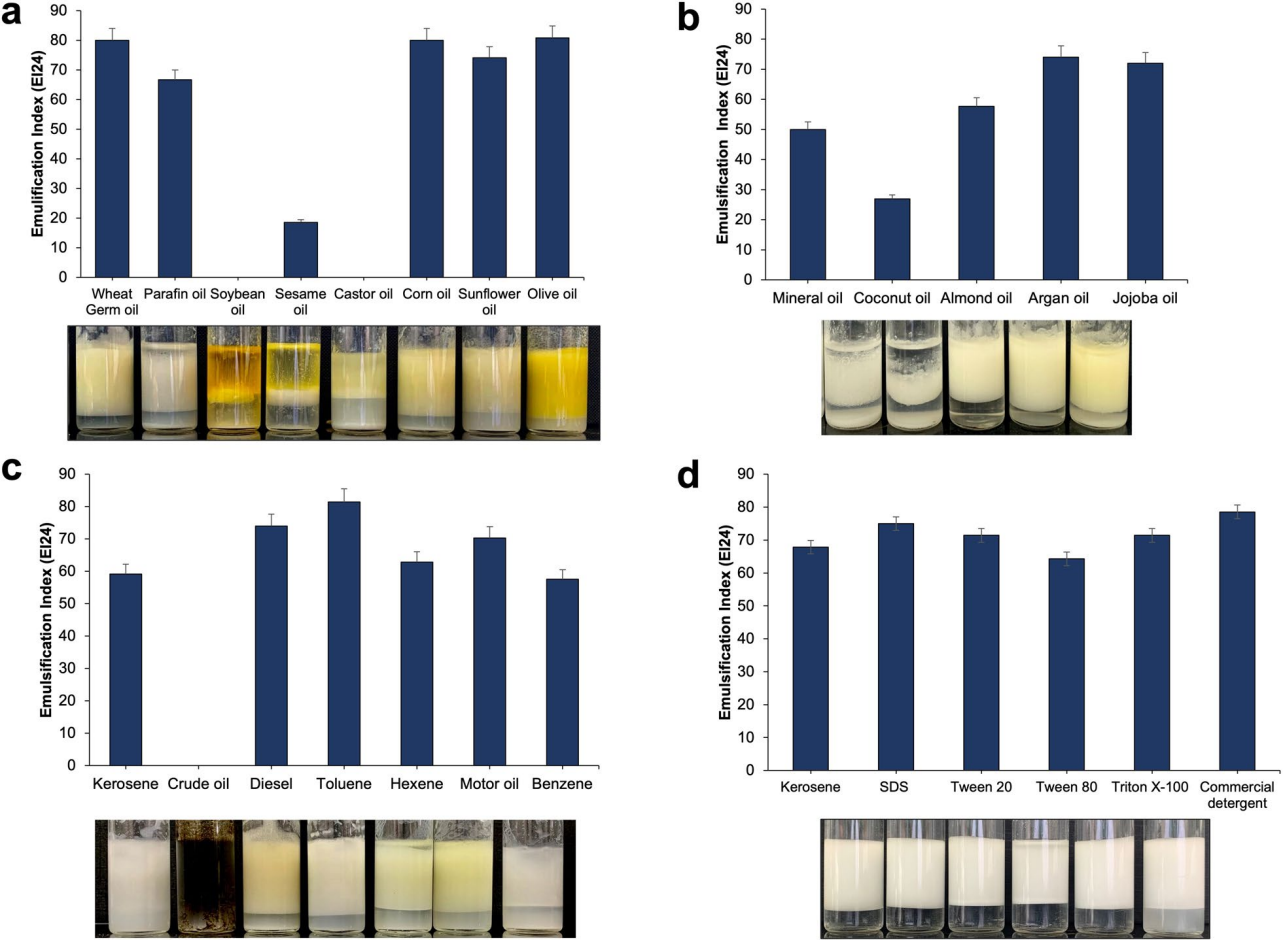
Emulsification index (EI_24_) of *S. cerevisiae* strain MYN04 bioemulsifier SC04 with different hydrophobic substrates after 24 h. **a** edible oils, **b** cosmetic oils, **c** hydrocarbons and **d** synthetic biosurfactants

The efficacy of the bioemulsifier was investigated with triglycerides, hydrocarbons, and a synthetic biosurfactant (Fig. 6). For edible oils, the highest EI_24_ values (80.0%) were recorded with wheat germ, corn, and olive oil substrates, while soybean and castor oil were not emulsified. Moreover, argan oil had the highest EI_24_ (74.0%) among cosmetic oils. Furthermore, bioemulsifier SC04 exhibited significant emulsification activity with different sources of hydrocarbons. Toluene had the highest EI_24_ (81.4%), while crude oil did not emulsify. On the other hand, bioemulsifier SC04 showed a high emulsification index (68.0%) compared to 78.0% for commercial detergent and was better than Tween 80 (64.0%) as a synthetic surfactant.

### Purification and characterization of bioemulsifier SC04

**Fig. 7:**
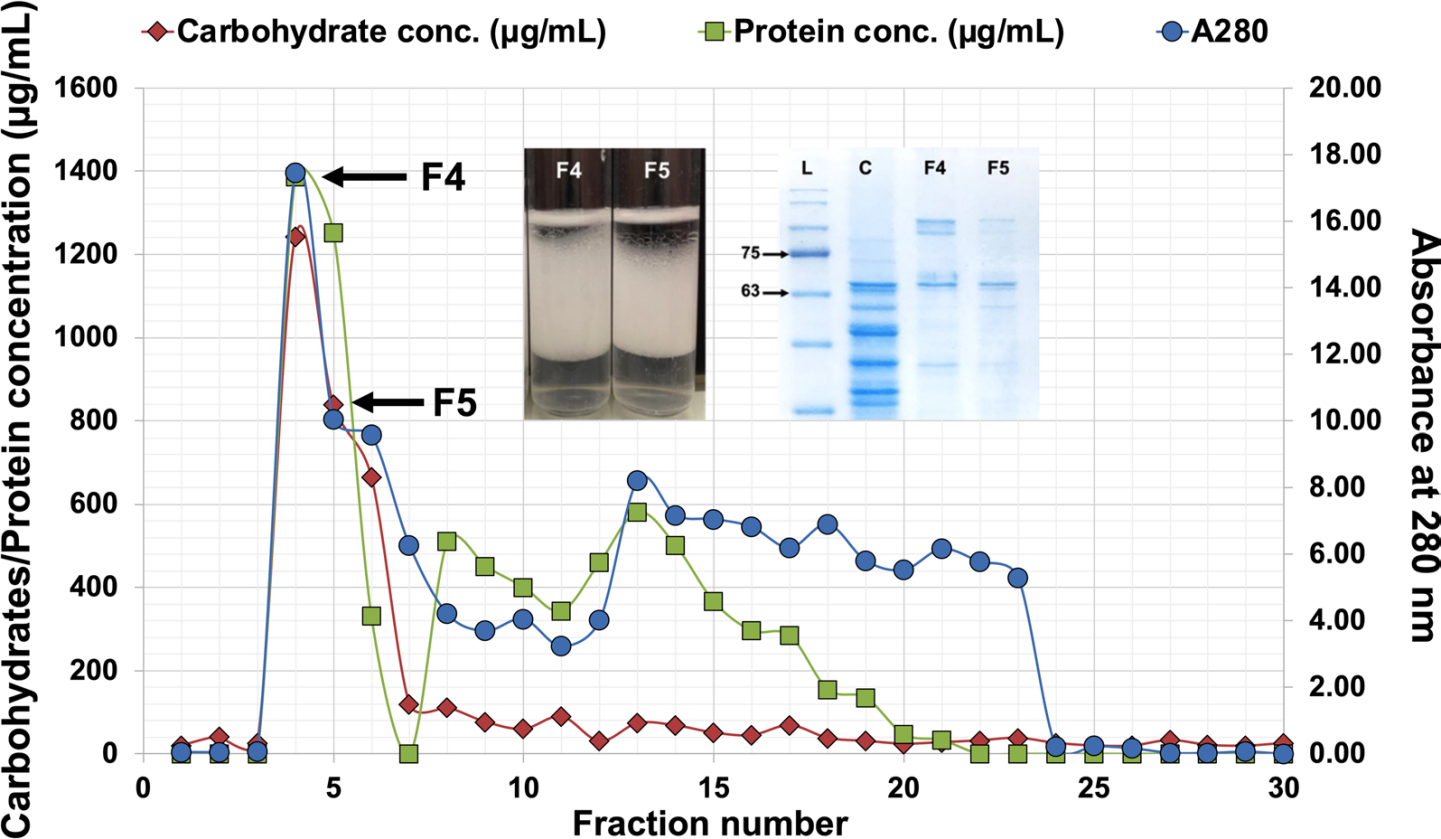
Gel filtration chromatography using Sephadex G-75. The column was eluted with 0.05 M potassium phosphate buffer (pH 7.0) at a flow rate of 5 mL/min. The presence of proteins in eluents was measured with UV‒Vis at A280, and the concentration (µg/mL) was measured with the Bradford method at A595. The concentration of carbohydrates (µg/mL) was measured with the Dubois method at A490

Bioemulsifier SC04 was precipitated with 50% ethanol and purified by fractionation based on molecular weight using Sephadex G-75. The elution profiles were examined for proteins (A_595_), carbohydrates (A_490_), and EI_24_%. Two fractions, F4 and F5, revealed significant emulsification activity and high carbohydrate and protein contents (Fig. 7). The SDS‒PAGE profile indicated that F5 was the highly purified fraction. Fraction 5 was dried by lyophilization and subjected to physicochemical characterization.

### Physicochemical characterization of bioemulsifier SC04

#### Structural functionalization by FTIR

**Fig. 8:**
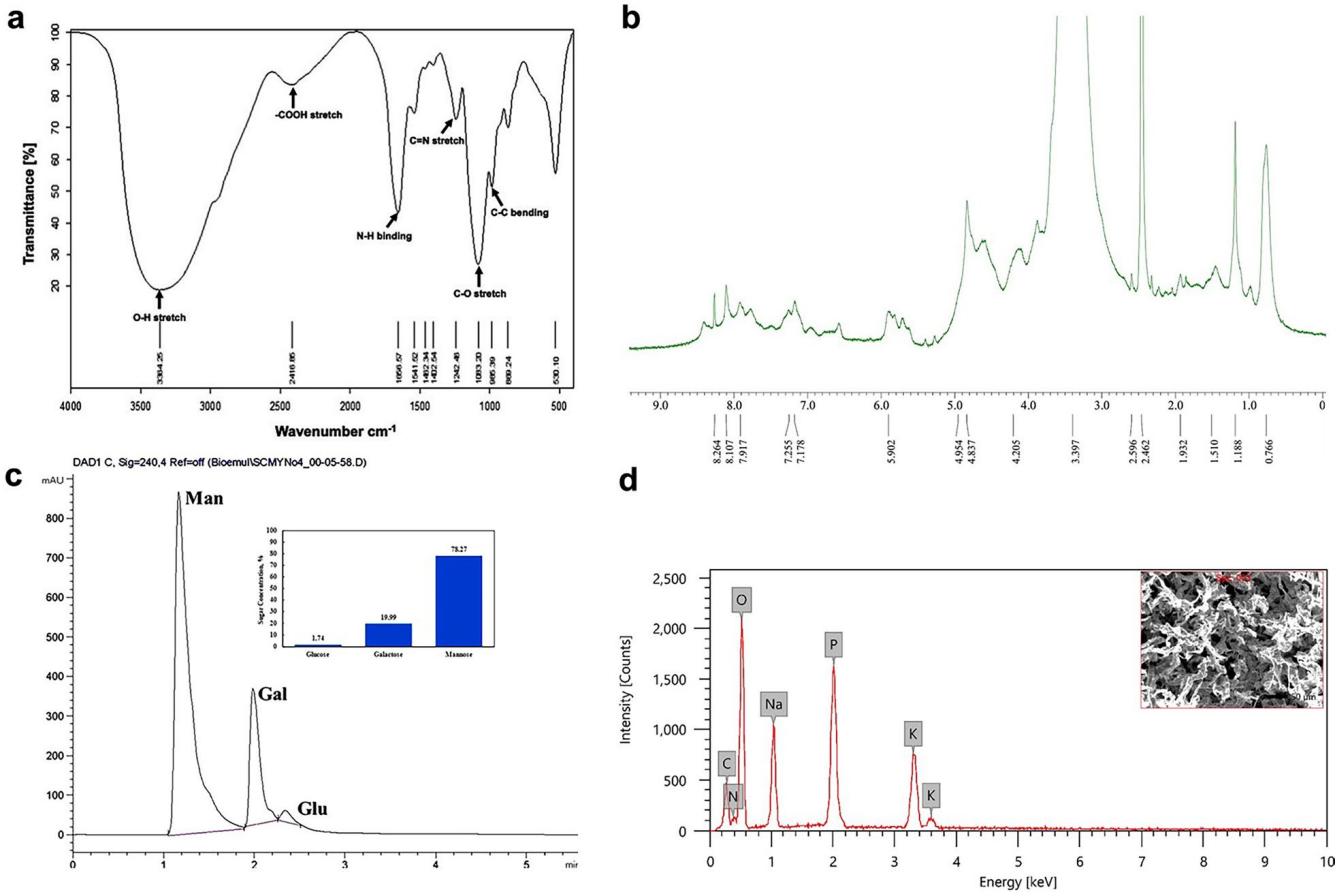
**a** FTIR spectrum, **b** ^1^H NMR spectrum obtained at 500 MHz, **c** HPLC chromatogram and **d** SEM-EDX analysis of bioemulsifier SC04

The structure of purified bioemulsifier SC04 was studied using various analytical methods. FTIR was used to identify the main functional groups in the bioemulsifier (Fig. 8a). The absorption peak at 3364.25 cm^−1^ corresponded to presence of the hydroxyl group (–OH) in the sugar moieties of carbohydrates. The presence of a peak at 2416.84 cm^−1^ revealed the presence of carboxylic group (–COOH). The absorptions at 1656.57 and 1541.5184 cm^−1^ corresponded to the C=O stretching of amide I and N–H bending in amide II, respectively. Additionally, bands at 1462.34 and 1402.54 cm^−1^ were associated with the C–H bend and CH_2_/CH_3_. The band at 1242.46 cm^−1^ corresponded to the C=N stretching of amine. The absorbance peaks at 1083.19 and 985.38 cm^−1^ corresponded to C–O stretching and C–C bending, respectively. Moreover, the absorption peak at 869.24 cm^−1^ was associated with the stretching vibrations of glycosidic linkages between sugar moieties. Finally, the absorption peak at 530.09 cm^−1^ indicated the presence of sugar derivatives.

### NMR analysis

The ^1^H NMR spectrum of bioemulsifier SC04 revealed the presence of R–CH_3_, R–CH_2_–R, and R_3_–CH alkane groups, which appeared in the proton range of δ = 0.7–2.0 ppm. Alcohol (R-OH) appeared in the range δ = 3.4–4.0 ppm. Furthermore, vinylic (R_3_C=CH) was predicted in the range δ = 4.5–6.0 ppm. Moreover, amides (R–CONH) appeared in the range δ = 5.5–8.5 ppm (Fig. 8b).

### Determination of monosaccharide composition by HPLC

The chromatographic analysis of the hydrolysed bioemulsifier revealed the presence of monosaccharides, including mannose, galactose, and glucose. Glucose was detected in minor concentrations compared to the other sugars. Monosaccharide peaks estimated by the HPLC method were observed in the chromatogram (Fig. 8c).

### Elemental analysis using EDX spectroscopy and morphological studies by SEM

**Fig. 9:**
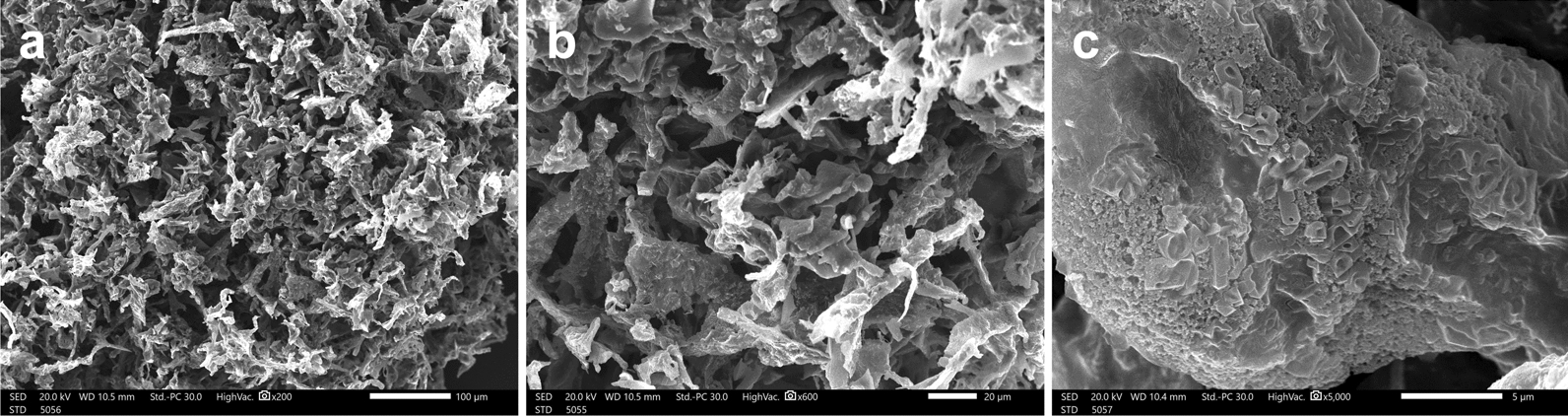
Scanning electron micrographs of bioemulsifier SC04 at **a** 200 ×, **b** 600 × and **c** 5000 × magnification

EDX was employed to assess the elements present in terms of weight%. The qualitative elemental analysis by EDX revealed the predominance of oxygen and carbon with mass ratios of 46.75 and 19.08 (w/w%), respectively (Fig. 8d). The mass ratio of phosphorus was 11.82 (w/w%), and that of nitrogen was 5.45 (w/w%). The SEM micrographs of bioemulsifier SC04 produced by the MYN04 strain revealed an irregular porous structure (Fig. 9).

### Antioxidant activity

The antioxidant activity of bioemulsifier SC04 was investigated in vitro using the DPPH assay. The percentage of DPPH scavenging was emulsifier dose dependent. At a concentration of 0.5 mg/mL of the bioemulsifier, the inhibition percent in the DPPH assay was found to be 51.8%, which was approximately 55.0% compared to the standard ascorbic acid at the same concentration. The IC_50_ value was estimated to be 386.4 µg/mL.

### Assessment of cytotoxicity and anticancer activity of the bioemulsifier

The cytotoxicity of SC04 was evaluated in vitro against normal epithelial kidney cell lines using the MTT assay. The bioemulsifier revealed a noncytotoxic effect against the tested cell lines, and the IC_50_ value was determined to be 393.2 ± 3.6 µg/mL. The cells showed a decrease in viability at concentrations higher than 250 µg/mL, indicating the safety of SC04 against normal kidney cells. Furthermore, SC04 exhibited anticancer effects against MCF7 breast cancer cell lines with an IC_50_ value of 202.4 ± 2.0 µg/mL. The toxicity of SC04 against breast cancer cells was approximately 70.5% using a concentration of 250 µg/mL. The toxicity of the bioemulsifier increased up to 93.0% by increasing the concentration up to 500 µg/mL.

## Discussion

In recent times, there has been a growing interest in the development of eco-friendly bioemulsifiers that possess high stability and emulsifying properties. These bioemulsifiers have the potential to replace synthetic surfactants for sustainable development in industries such as food, pharmaceuticals, and bioremediation of oil-contaminated wastes [7, 15]. Previous studies have reported that yeast cell wall mannoproteins are functionally associated with emulsification properties [13, 14]. In this study, *S. cerevisiae* strain MYN04 was selected for the production and optimization of the eco-friendly bioemulsifier SC04. Furthermore, the new bioemulsifier, SC04, was subjected to physicochemical characterization and applications.

The emulsification index (EI_24_) was used as a selection criterion for potential bioemulsifier-producing strains [16]. Hence, the primary selection of a potent bioemulsifier-producing strain was based on its capability to form an oil-water emulsion [17]. Moreover, the intracellular extract of the MYN04 isolate exhibited the most significant emulsification index and biomass, which was approximately two times higher than that obtained with *Meyerozyma caribbica* [18]. The isolate was identified by molecular techniques as *S. cerevisiae* strain MYN04. Previously, *S. cerevisiae* 2031 [16] and *S. cerevisiae* URM 6670 [7] have been reported to form stable emulsions with different oils.

The growth kinetics of *S. cerevisiae* MYN04 revealed that the production of the bioemulsifier was growth-associated and reached its maximum during the stationary phase. Similar results were reported for different microorganisms [18–20]. The maximum production of bioemulsifiers by *Parapedobacter indicus* was also recorded in the stationary phase [21]. The optimization of bioemulsifier production is a significant step to maximize yield, which is ultimately subjected to in-depth characterization and biological applications. The production yield varies according to substrate composition, production strain, and cultivation parameters [22]. In this experiment, the optimization of the medium was carried out by changing one factor at a time. The results revealed that the production of the bioemulsifier was highly dependent on the carbon and nitrogen sources (data not shown). In terms of the carbon source, sucrose achieved the highest CDW, followed by glucose as the sole carbon source. Different studies have reported glucose as the best carbon source for the production of surface-active compounds [18, 23]. It has also been reported that microorganisms mostly utilize glucose due to its easier metabolism via the glycolytic pathway for the generation of desired metabolites [24]. Some studies have revealed that metabolic pathways involved in bioemulsifier biosynthesis are regulated by the sugar used as a carbon source during growth [25, 26]. Complex nitrogen sources, such as yeast extract and peptone, are important nitrogen sources for cell growth. In the present study, maximum CDW and EI_24_ were obtained with yeast extract, followed by peptone as the sole nitrogen source. Similar results were reported for biosurfactant production using yeast extract as a nitrogen source [27]. Other studies have reported ammonium salts, including ammonium sulphate [18] and ammonium nitrate [9], as the best nitrogen sources. The differences in the utilization of carbon and nitrogen sources could be attributed to the differences in organisms and growth conditions.

The use of statistically designed experiments is increasingly replacing the one-variable-at-a-time (OVAT) approach [28, 29]. Classical methods are limited by cost, time, and resource requirements and their inability to predict interactions between variables. In contrast, statistically designed experiments are favoured due to their ability to overcome these limitations [30, 31]. PB design experiments revealed a 64.6% increase in EI_24_, which is 1.1-fold higher than that of the OVAT approach. Sucrose, FeSO_4_, volume of medium, and yeast extract were significant variables. Yeast extract had a similar negative effect on biosurfactant production using *Bacillus subtilis* and *Yarrowia lipolytica* [28, 32]. Increasing the concentration of sucrose and CaCl_2_ had negative effects; however, slight positive effects for both variables were reported for the production of biosurfactant by *Bacillus* sp. DSW17 [33]. Additionally, FeSO_4_ showed a comparable positive effect for *S. cerevisiae* strain MYN04 and *Bacillus* sp. DSW17 [33]. Previous studies have suggested that iron plays an important role in the regulation of genes involved in bioemulsifier synthesis at the transcriptional level [34]. An increase in inoculum size resulted in a decrease in emulsification activity and EI_24_ due to nutrient depletion for elevated metabolic activities [21]. The optimal inoculum size for this strain was approximately 1.5%, which is close to the reported size of 2% (v/v) [21, 35].

Response surface methodology (RSM) is frequently used to develop emulsifying agents, including CCD and PBD [36]. RSM is significant in revealing the interaction of variables and their effect on the process response [37]. For further optimization using the CCD, sucrose concentration, FeSO_4_, and pH were chosen for their effect on bioemulsifier biosynthesis indicated by EI_24_, %. The medium volume of 75.0 mL was optimal for better aeration, and CaCl_2_, MgSO_4_, KH_2_PO_4_, and yeast extract were used at their low levels (0.2, 2.5, 2.0, and 5.0 g/L, respectively) due to their negative effect on the process, while inoculum size was applied at its middle level (1.5%, v/v) as it had a positive effect. High EI_24_ values (72.0, 65.2%) were observed at initial pH values of 6.5 to 7.5. Maximum emulsification activities were reported for pH 7.0 [21, 38, 39]. The production of bioemulsifiers and biosurfactants is generally near neutral pH, although some reports suggest a slight increase in pH from 7.0 to 8.5 [40, 41]. Variation of sucrose concentration as the sole source of carbon showed that the optimal range for emulsification activity was 20.0 to 26.0 g/L. Biosurfactant production was reported to be closely associated with the growth of *Bacillus* sp. DSW17 and *Pseudomonas fluorescens* [33]. It is worth mentioning that many studies have reported the use of sugars alone or combined with oily substrates [33, 36, 42, 43]. FeSO_4_ showed a recognizable positive effect on bioemulsifier biosynthesis by *S. cerevisiae* strain MYN04. A slight reduction in emulsifying activity was observed by increasing the concentration of FeSO_4_ from 0.05 to 0.08 g/L (Table 2). A lower concentration of FeSO_4_ (0.014) was reported for the production of surface-active rhamnolipids by *P. aeruginosa* AT10 [44] compared to 0.05 g/L for bioemulsifier biosynthesis by *S. cerevisiae* strain MYN04. The maximum CDW was obtained at 50.0 g/L, 7.5, and 0.03 g/L of sucrose, pH, and FeSO_4_, respectively. The change between initial and final pH values (ΔpH) was measured, and the maximum difference was reached at -1.6 at an initial pH of 8.0 (data not shown).

The quadratic model effectively described the relationships between variables and responses. Sucrose concentration, its interactions, and FeSO_4_ significantly affected EI_24_, %, while pH and its interaction with FeSO_4_ were found to be insignificant. The quadratic model showed high accuracy within the experimental levels of the CCD in predicting optimized EI_24_, % (Table 3). The predicted value at sucrose concentrations of 26.0 g/L, 0.07 g/L FeSO4, and pH 6.5 was only off by 1.5 × 10^−5^%. However, the third run was selected at 12.0 g/L sucrose, which was not included in the tested domain and resulted in the maximum EI_24_ in this study (79.0 ± 2.0%), which falls within the predicted range (77.6–82.3%) with 95% confidence. The quadratic equation is as follows:

$$\begin{aligned} {\text{E}}24,\% {\text{ = }} & {\text{199}}.{\text{0 + 0}}.{\text{17X}}_{{\text{1}}} {\text{ + 1477}}.{\text{1X}}_{{\text{2}}} {\text{ + 66}}.{\text{2X}}_{{\text{3}}} {\text{ - 12}}.{\text{4X}}_{{\text{1}}} {\text{X}}_{{\text{2}}} \\ {\text{ - }} & {\text{0}}.{\text{2X}}_{{\text{1}}} {\text{X}}_{{\text{3}}} {\text{ - 26}}.{\text{0X}}_{{\text{2}}} {\text{X}}_{{\text{3}}} {\text{ + 0}}.{\text{02X}}_{{\text{1}}} ^{{\text{2}}} {\text{ - 6767}}.{\text{82X}}_{{\text{2}}} ^{{\text{2}}} {\text{ - 4}}.{\text{34X}}_{{\text{3}}} ^{{\text{2}}} \\ \end{aligned}$$

where X_1_ is the sucrose concentration, X_2_ is the concentration of FeSO_4_, and X_3_ is the initial pH of the cultivation medium.

The pairwise correlations between responses of the CCD (CDW, EI_24_, and ΔpH) indicate that CDW was significantly and linearly correlated (0.949–*P* = 0.000) with sucrose concentrations (Table 4). The correlation was at its lowest value for CDW and FeSO_4_, followed by EI_24_-pH and ΔpH-EI_24_. Sucrose was moderately inversely correlated with EI_24_ (− 0.525, *P* = 0.004). Interestingly, the relationship between EI_24_ and CDW of *S. cerevisiae* strain MYN04 is a moderate inverse linear correlation (− 0.47, *P* = 0.012).

**Table 4:** Pairwise Pearson correlations between responses and variables investigated for optimization of the biosynthesis of bioemulsifier by *S. cerevisiae* strain MYN04 using CCD

Pairs	Correlation	*P* value
CDW-Sucrose	0.949	0.000
CDW-pH	0.196	0.317
CDW-FeSO_4_	0.087	0.660
EI_24_-Sucrose	− 0.525	0.004
EI_24_-pH	0.099	0.616
EI_24_-FeSO_4_	0.408	0.031
EI_24_-CDW	− 0.47	0.012
ΔpH-pH	− 0.729	0.000
ΔpH-EI_24_	0.115	0.559
ΔpH-Sucrose	− 0.33	0.087
ΔpH-CDW	− 0.485	0.009

*CDW* cell dry weight, *ΔpH* difference between initial and final pH

The relevance of bioemulsifiers in different fields depends on their stability at various temperatures, pH values, and salinities. The results showed that bioemulsifier SC04 was stable in the tested temperature and pH range and remained constant for at least one month at different pH values, indicating its suitability for industrial applications (Fig. 5). The present bioemulsifier could be more suitable for application than that obtained by *Kluyveromyces marxianus* FII 510,700, which showed no emulsification at pH 2.0 and retained only approximately 70% of the activity at pH up to 11.0 [45]. On the other hand, the bioemulsifier from *S. cerevisiae* 2031 was found to be sensitive in the pH range of 4.0–6.0 and stable in the acidic and alkaline range [46]. Many references have cited the stability of bioemulsifiers from different organisms [5, 18, 47]. Bioemulsifier SC04 was also stable in different NaCl concentrations between 2 and 10% (w/v) for at least two weeks at room temperature, suggesting its halotolerance. Due to its stability in the presence of high salt concentrations, bioemulsifier SC04 could be useful for the bioremediation of oil spills in the marine environment [48]. It is also better than chemical surfactants such as SDS, Triton X-100 or Tween 80, which do not show emulsifying activity at NaCl concentrations of 10–12% [49].

The capacity of bioemulsifiers to stabilize emulsions has increased their applications in industries such as food, pharmaceuticals, and petroleum. The results of our study revealed that bioemulsifier SC04 efficiently emulsified various edible and cosmetic oils, with the exception of soybean and castor oil, which were not emulsified. Olive, corn, and sunflower oils were emulsified at a degree higher than that reported in other studies [46, 50]. The bioemulsifier also emulsified different hydrocarbons with an emulsification index ranging from 58.0 to 81.0%, except for crude oil (Fig. 6). The emulsification of various hydrocarbons with different emulsification indices has been reported in many studies [46, 51, 52]. Some hydrocarbons were poorly emulsified, possibly due to the inability of the bioemulsifier to stabilize the microscopic droplets [51]. The ability of the produced bioemulsifier to emulsify different hydrocarbons is essential for its use in the treatment of industrial effluents. The study also revealed a good emulsification index for bioemulsifier SC04 compared to that of synthetic surfactants, which is consistent with similar studies [46].

The bioemulsifier was precipitated by ethanol, and a gel filtration column was used for fractionation. Two fractions (F4 and F5) were identified with positive results for emulsification, which were then lyophilized and evaluated for total sugar, proteins, and emulsifying activity. The lyophilized bioemulsifier SC04 had a white, fluffy texture, while the bioemulsifier produced by *Parapedobacter indicus* was off-white and had an amorphous, powdery nature after lyophilization [21]. Ethanol has been used for the precipitation of various bioemulsifiers [21, 46] and gel filtration has been used as a tool for the purification of bioemulsifiers/biosurfactants in many studies [18, 46]. The protein part was confirmed by SDS‒PAGE, which revealed high purity and high molecular weight of the bioemulsifier. Studies have shown that high molecular weight biosurfactants are generally better emulsifiers than low molecular mass biosurfactants [53].

This investigation aimed to characterize the *S. cerevisiae* MYN04-derived bioemulsifier. The chemical composition of the bioemulsifier revealed that it is a mixture of 27.1% carbohydrates and 72.9% proteins that are likely bonded to form a complex. These results are consistent with those obtained for the bioemulsifier produced by *Parapedobacter indicus* [21] but contradictory to those obtained for yeasts, in which the carbohydrate moiety represents a high proportion ranging from 50 to 90% [18, 54]. Previous studies have revealed that the lipophilic moiety of the bioemulsifier has significant emulsification activity and could be a protein with a high proportion of hydrophobic side chains [55]. Additionally, the carbohydrate moiety is responsible for the stability of the emulsion [54]. Moreover, FTIR analysis revealed the presence of basic hydroxyl and amine functional groups, indicating the possible carbohydrate and protein nature of the bioemulsifier. These results are partially consistent with those obtained for the bioemulsifiers produced by *Parapedobacter indicus* [21], strain *S. silvestris* AM1 [56], and *Acinetobacter beijerinckii* ZRS [57], while they are similar to those obtained for *S. cerevisiae* 2031 [46].

To obtain structural information on bioemulsifier SC04, ^1^H NMR analysis was performed. Chemical shift (δ) values at 1.11–1.20 ppm indicated the presence of the methyl group (CH_3_) corresponding to the sugar moiety, while values at 4.8 and 4.9 indicated the presence of protons on the β-anomeric carbon of sugar moieties. Additionally, the amino sugar moiety resonated in polymeric surfactants at δ = 7.0-8.5 ppm [58]. Moreover, the EDX results revealed the presence of oxygen, carbon, and nitrogen, which are in agreement with the FTIR results, indicating that the bioemulsifier is mainly composed of carbohydrates and protein. The predominance of oxygen and carbon in bioemulsifier SC04 represents carbonyl functional groups, as previously described [20, 59]. The presence of phosphorus in bioemulsifier SC04 may be due to elution of the sample from the column with phosphate buffer.

The monosaccharide composition of bioemulsifier SC04 revealed the presence of approximately 78% mannose, along with approximately 20% galactose and traces of glucose. These results are consistent with those reported for bioemulsifiers with a high content of mannose produced by different yeast strains [7, 13, 14]. Studies have shown that mannose and protein in the emulsifier are necessary for its action as an emulsifier. Furthermore, the presence of hydrophilic mannose attached to the protein backbone provides the mannoprotein with an amphiphilic structure common to surface-active agents [54].

Antioxidant activity is a desirable property for substances to be incorporated into industrial applications [7]. The DPPH radical-scavenging assay was used to study the ability of bioemulsifier SC04 to act as a free radical scavenger or hydrogen donor. Our results showed better DPPH radical scavenging activity compared to that obtained with the biosurfactant obtained from *S. cerevisiae* URM 6670 [7], who reported approximately 13% inhibition in DPPH using 20 mg/mL biosurfactant. Our results were also better than those reported by [60], who found that the bioemulsifier produced by the yeast *Pseudozyma hubeiensis* exhibited 50% DPPH radical scavenging activity but at a higher concentration (10 mg/mL). Additionally, it was reported that the DPPH scavenging activity of biosurfactants produced by *Lactobacillus casei* was between 74.6 and 77.3% at 5.0 mg/mL [61].

*S. cerevisiae* is a food-grade yeast widely used in the food industry. Therefore, the emulsifier would be expected to be nontoxic [7]. However, evaluating bioemulsifier toxicity before proposing them in food industries is important to confirm their safety. In the present study, the MTT results revealed that the bioemulsifier could be used in food applications, as it exhibited no cytotoxic effect against normal epithelial kidney cells at concentrations up to 250 µg/mL. Our results were in accordance with previous work by [7], who reported no risk of toxicity of the biosurfactant produced by *S. cerevisiae* URM 6670 at the tested concentration of 200 µg/mL. However, further in vivo tests are required to confirm the safety of bioemulsifier SC04. The cytotoxicity assay in BHK-21 cell lines revealed 63% cell survival at 10,000 µg/mL for the biosurfactant produced by *Bacillus cereus* [62], and the author reported it as safe. Furthermore, bioemulsifier SC04 exhibited an anticancer effect against MCF7 breast cancer cell lines. A previous study revealed that injection with heat-killed non-pathogenic *S. cerevisiae* induced highly significant levels of apoptosis in human breast cancer-bearing mice [63]. The anticancer activity could be associated with the cell wall components, mainly mannoproteins. The study reported by [64] revealed that biosurfactants are involved in several intracellular molecular recognition steps, including signal transduction, cell differentiation and cell immune response.

## Conclusion

This study reported on a new bioemulsifier, SC04, produced by the *S. cerevisiae* strain MYN04. Chemical characterization of the bioemulsifier revealed that it is a high molecular weight mannoprotein complex. Furthermore, the bioemulsifier SC04 was able to form stable emulsions with a variety of oils and persisted upon exposure to extreme environmental conditions. These features of bioemulsifier SC04 indicate its potential for a variety of industrial and environmental applications.

## Materials and Methods

### Isolation of yeast and screening for bioemulsifier production

Baker’s fresh and dried yeast samples were collected from the local Egyptian market and isolated using the serial dilution method on yeast malt agar plates (YM; Difico). The plates were incubated at 30 °C for 48 h. The isolates were purified using the streak plate method [65], and pure colonies were selected according to cell morphology using light microscopy and stored in 50% glycerol at − 20 °C for further investigation. YM broth flasks (100 mL) were inoculated with 2% (v/v) overnight cultures and incubated statically at 30 °C for 48 h. Cultures were centrifuged at 3000 rpm and 4 °C for 10 min. Then, the cell pellets were mixed with 5 mL of potassium dihydrogen phosphate buffer (pH 7.0; Merck, Germany), subjected to sonication (Sonopuls, Germany) for 5 min, and centrifuged at 3000 rpm, and the supernatant was used as a source of crude emulsifier [66]. The emulsification index (EI_24_) was determined by adding 4 mL of the supernatant to 6 mL of kerosene, vigorously mixing by vortexing at high speed for 2 min and allowing the mixture to stand for 24 h. The emulsification activity was determined as a percentage using the following equation: EI_24_ = Height of emulsion formed x 100/Total height of solution [16].

###  Molecular identification of selected strain

DNA was extracted from the potent yeast strain using the Quick-DNA™ Fungal/Bacterial Miniprep Kit (Cat # D6005; ZymoResearch, USA) following the manufacturer’s instructions. Purified DNA was used as a template for 26S rRNA gene amplification using universal primers. The DNA polymerase COSMO PCR RED Master Mix (Cat # W1020300X, Willowfort, UK) was used for DNA amplification in a final volume of 50 µL containing: 25 µL premix, primers at concentration 20 µM, 2 µL DNA template (100 ng/µL) and up to 50 µL nuclease-free H_2_O. The PCR conditions were as follows: initial denaturation at 95 °C for 2 min, denaturation at 95 °C for 15 s, followed by annealing for 1 min, extension at 72 °C for 30 s, and final extension at 72 °C for 1 min for 35 cycles. The 26S rRNA amplicon for the selected isolate was analysed using the ABI 3730XL DNA Analyser (ACGT, Germany). Matching and similarity were detected using the NCBI (National Centre for Biotechnology Information) Basic Local Alignment Search Tool (BLAST) database (https://blast.ncbi.nlm.nih.gov). For phylogenetic analysis, the 26S large subunit rRNA sequences were downloaded from the NCBI database, followed by alignment using ClustalW (v 2.0.3), phylogeny using PhyML (v 3.0), and tree rendering by TreeDyn (v 198.3). The previously listed programs were used through phylogeny.fr (https://www.phylogeny.fr/) [67].

### Optimization of bioemulsifier production

#### Influence of different carbon and nitrogen sources

Isolate MYN04 was cultivated using different carbon and nitrogen sources. Cooper and Paddock’s medium was supplemented with glucose, maltose, fructose, xylose, raffinose, and sucrose as sole carbon sources and yeast extract, peptone, casein, ammonium chloride, ammonium nitrate, ammonium sulphate, and ammonium orthophosphate as sole nitrogen sources. The incubation was performed on a shaker incubator at 30 °C and 150 rpm for 36 h. At the end of the experiment, the cells were harvested by centrifugation at 4000 rpm and 4 °C. The cell dry weight (g/L) and EI_24_ were determined. This experiment was replicated.

#### Exploration and optimization of Emulsifier Biosynthesis

A preliminary survey was conducted to inspect the variables that affect the growth and biosynthesis of bioemulsifiers by *S. cerevisiae* strain MYN04 using the Plackett‒Burman design (PB). The significant influencing factors can be found in Table 5, where 10 medium components were selected. The responses selected for evaluation were cell dry mass and the percentage obtained from the emulsification tests. The selected variables were subsequently subjected to a central composite design (CCD) to determine the optimum values for bioemulsifier activity. The design integrated three levels for each variable and six centre points for the studied factors (Designer expert 12, Stat-Ease Inc., Minneapolis, USA), as shown in Table 6. In experiments involving three variables, the mathematical relationship of the response (EI_24_) with the chosen variables was estimated by the quadratic polynomial equation given below:

**Table 5:** List of factors and levels of the PB

Factors	Units	Levels	
		− 1	+ 1
Sucrose	g/L	15.0	25.0
Yeast Extract	g/L	3.0	7.0
Potassium dihydrogen Phosphate	g/L	0.5	1.5
Magnesium Sulphate	g/L	2.5	7.5
Calcium Chloride	g/L	0.1	0.3
Sodium Chloride	g/L	0.1	1.0
Ferrous Sulphate	g/L	0.01	0.04
Inoculum size	%	1.0	3.0
Medium’s volume	mL	75.0	125.0
pH	Unit	3.0	7.0

− 1, low level; + 1, high level

**Table 6:** List of factors and levels of the CCD

Factors	Units	Levels		
		− 1	0	+ 1
Sucrose	g/L	26.0	38.0	50.0
Ferrous sulphate	g/L	0.03	0.05	0.07
pH	Unit	5.5	6.5	7.5

$$\begin{aligned} Y = \,& a_{0} + a_{1} X_{1} + a_{2} \;X_{2} + a_{{3\;}} X_{3} + a_{{12}} \;X_{1} \;X_{2} + a_{{13}} \;X_{1} \;X_{3} \\ + & a_{{23}} \;X_{2} \;X_{3} + a_{{11}} X_{1} ^{2} + a_{{22}} X_{2} ^{2} + a_{{33}} X_{3} ^{2} \\ \end{aligned}$$

where Y is the response value; a_0_ is the constant; a_1_, a_2_, and a_3_ are the linear coefficients; a_12_, a_13_, and a_23_ are the interaction product coefficients; and a_11_, a_22_, and a_33_ are the quadratic coefficients.

Finally, experiments were performed to verify the reliability of the chosen experimental model and prediction of optimized conditions. All experiments were carried out in triplicate, and the results are presented as the average values. Pairwise Pearson correlation was performed on the average of responses using Minitab (2019) Statistical Software, Version 19 (Minitab Inc., State College, USA).

#### Large-scale production and isolation of crude bioemulsifiers

The growth of *S. cerevisiae* strain MYN04 was performed in a batch fermenter containing 10 L of production medium for 36 h. To recover the bioemulsifier, cells were separated by centrifugation at 3000 rpm and 4 °C for 20 min. The cells were suspended in potassium dihydrogen phosphate buffer (pH 7.0; Merck, Germany) and subjected to sonication. The supernatant was treated with cold ethanol until precipitation formed and then collected by centrifugation at 3000 rpm and 4 °C for 20 min.

#### Stability studies of the crude bioemulsifier

The impact of temperature on the emulsifier activity was investigated by keeping the bioemulsifier at 4, 30, 50, 70, 90, and 121 °C for 60 min. The effect of salinity was studied by exposing the bioemulsifier to different concentrations of NaCl (2, 4, 6, 8, and 10% [w/v] NaCl). The impact of pH on stability was tested by incubating the bioemulsifier at different pH values (2, 4, 6, 8, and 10). All treatments were assessed by calculating EI_24_.

#### Assessment of emulsification activity of the bioemulsifier

The emulsification abilities were investigated against different edible oils (wheat germ, paraffin, soybean, sesame, castor, corn, sunflower, and olive oil), cosmetic oils (mineral, coconut, argan, almond, and jojoba oil), different hydrocarbons, and synthetic biosurfactants (SDS, Tween 20, Tween 80, and Triton X-100). The bioemulsifier was mixed with an equal volume of each oil and vortexed for 2 min, and EI_24_ was determined after settling for 24 h.

### Purification and physicochemical characterization of bioemulsifier

#### Total carbohydrate and protein content

The total carbohydrate and protein contents of the purified bioemulsifier were assessed using the phenol sulphuric acid method [68] and Bradford assay [69], respectively. The carbohydrate reaction mixture was measured at an absorbance of 490 nm, while the protein reaction mixture was measured at an absorbance of 595 nm using UV‒Vis spectrophotometer (Jenway 6305, UK). Standard plots were prepared using glucose (10–100 µg/mL) and bovine serum albumin (0.5–2.5 mg/mL).

#### Size exclusion chromatography

The crude bioemulsifier sample was subjected to further purification using a gel filtration chromatography column packed with Sephadex G-75 (Acros Organics, Germany). The column was washed and eluted with 0.05 M potassium phosphate buffer (pH 7.0) at a flow rate of 5 mL/min. The collected fractions were measured at an absorbance of 280 nm using UV‒Vis spectrophotometer (Jenway 6305, UK), and the total protein content, total carbohydrates, and total emulsification activity (EI_24_) were determined. Fractions with higher emulsification activity were pooled and lyophilized in a freeze dryer (CHRIST, Germany) and stored for further characterization.

#### Polyacrylamide gel electrophoresis (SDS‒PAGE)

The presence of protein moieties in the bioemulsifier was validated using 12% tris-glycine SDS‒PAGE [70]. The bioemulsifier fractions were resuspended in denaturation buffer and incubated for 10 min at 95 °C. A prestained protein ladder (Maestrogen, China) of size 10–170 kDa was used as a standard.

#### Fourier transform infrared spectroscopy (FTIR) analysis

The freeze-dried pure bioemulsifier was subjected to FTIR spectrophotometer (Bruker Tensor 27, Germany) to identify the structural functionalities. The FTIR spectra were recorded at room temperature and in the frequency range of 4000 − 500 cm^−1^.

#### Scanning electron microscopy (SEM) and energy dispersive X-ray spectroscopy (EDX)

The purified bioemulsifier surface morphology and EDX elemental analysis were examined with scanning electron microscopy (SEM; JSM-IT 200, JEOL) at the EM Unit, Alexandria University, Egypt. Lyophilized SC04 (10 mg) was subjected to SEM visualization at an accelerating voltage of 20.0 kV [71].

#### Determination of monosaccharide composition using high performance liquid chromatography (HPLC)

The monosaccharide composition of the purified bioemulsifier SC04 (13 mg) was determined after hydrolysis with 4 M trifluoroacetic acid (TFA) at 124 °C for 6 h. The hydrolysate was evaporated at 40 °C and then dissolved in 3 mL of deionized water for monosaccharide composition analysis using HPLC (Agilent 1260, USA). The sample was injected into an HPLC column (C18), and the column effluent was monitored using a refractive index detector (RI, 2410). The UV detection wavelength was 243 nm. The mobile phase contained acetonitrile (75%) and 0.1% formic acid (25%) with a 1.8 mL/min flow rate [72]. The identification and quantification of monosaccharide profiles were performed using Breeze QS HPLC system software (Central Lab, Faculty of Science, Alexandria University).

#### Structural studies using nuclear magnetic resonance spectroscopy (NMR)

For structural studies of purified bioemulsifier SC04, proton nuclear magnetic resonance was performed at 500 MHz (^1^H NMR; JNM-ECZ500R, JEOL). Approximately 20 mg of lyophilized SC04 was completely dissolved in DMSO-D6. The ^1^H NMR spectrum was recorded, and the chemical shifts were expressed in ppm [73].

### Biological properties of bioemulsifier SC04

#### Evaluation of antioxidant activity by DPPH Assay

The free radical scavenging activity of the bioemulsifier was measured using 1,1-diphenyl-2-picryl hydrazyl (DPPH) [74]. Briefly, 0.1 mM DPPH solution was prepared in ethanol, and 1 mL was added to different concentrations of bioemulsifier (1000, 500, 250, 125, 62.5 and 31.25 µg/mL). The mixtures were shaken and incubated for 30 min at room temperature, and then the absorbance was measured at 517 nm using a spectrophotometer. The IC_50_ value was calculated using a log dose inhibition curve. The percent DPPH scavenging effect was calculated using the following equation: DPPH scavenging effect (%) = A0−A1/A0 × 100, where A0 was the absorbance of the control reaction and A1 was the absorbance in the presence of the test or standard sample. This experiment was performed in triplicate, and ascorbic acid was used as a reference standard.

#### Evaluation of cytotoxicity and anticancer activity

The bioemulsifier cytotoxic effect was evaluated using a 3-[4,5-dimethylthiazole-2-yl]-2,5-diphenyltetrazolium bromide (MTT) assay [75] against normal epithelial kidney cell lines (ATCC CCL-81) and the MCF7 breast cancer cell line (ATCC HTB-22). Cell lines (10^5^ cells/mL) were maintained in a 96-well tissue culture plate and incubated at 37 °C for 24 h. A two-fold dilution of the crude bioemulsifier was prepared in Roswell Park Memorial Institute medium (RPMI; Sigma‒Aldrich, USA) supplemented with 2% serum, and 100 µL of each dilution was tested. Then, 20 µL of MTT solution (5 mg/mL) in phosphate-buffered saline (PBS) was added to each well and mixed at 150 rpm for 5 min. Plates were incubated at 37 °C in a moist atmosphere enriched with 5% CO_2_ for 4 h to allow the metabolism of MTT. Plates were washed and allowed to air dry. Absorbance was measured at a wavelength of 560 nm and subtracted from the background at 620 nm.

## Data Availability

All data produced during this study are included in the article.

## Abbreviations

CCD: Central composite design
CDW: Cell dry weigh
DPPH: 1,1-diphenyl-2-picrylhydrazyl
EDX: Energy dispersive X-ray spectroscopy
EI_24_: Emulsification index
FTIR: Fourier transform infrared spectroscopy
GRAS: Generally recognized as safe
HPLC: High-performance liquid chromatography
IC_50_: Half maximal inhibitory concentration
MEOR: Microbial enhanced oil recovery
NCBI: National Center for Biotechnology Information
NMR: Nuclear magnetic resonance
OVAT: One variable at a time
PB: Plackett‒Burman
PBS: Phosphate buffered saline
RSM: Response surface methodology
SDS‒PAGE: Sodium dodecyl sulphate-polyacrylamide gel electrophoresis
SEM: Scanning electron microscopy
TFA: Trifluoroacetic acid
YM: Yeast malt
